# A model to quantify the probability of collision between birds and aircraft: Applications for onboard lighting

**DOI:** 10.1002/eap.70227

**Published:** 2026-04-01

**Authors:** Ryan B. Lunn, Bradley F. Blackwell, Esteban Fernández‐Juricic

**Affiliations:** ^1^ Department of Biological Sciences Purdue University West Lafayette Indiana USA; ^2^ United States Department of Agriculture Animal and Plant Health and Inspection Services, National Wildlife Research Center Sandusky Ohio USA

**Keywords:** animal and vehicle collisions, bird and aircraft collisions, Canada geese, escape behavior, model, probability of collision

## Abstract

Globally, bird and aircraft collisions are a major safety hazard and monetary expense for the aviation industry. Empirical evidence suggests that the behavioral response of an animal within close proximity of an approaching vehicle is a critical factor in determining whether a collision occurs. However, no theoretical framework exists to predict the probability of a collision based on the escape response of the animal to an approaching vehicle. We adapted concepts from existing predator–prey theoretical frameworks to develop a novel model to quantify the outcome of an animal–vehicle interaction. Specifically, our model consists of two distinct phases. Phase one determines if a collision is even possible based on the amount of time the animal has available to clear the trajectory of the approaching vehicle. If the animal does not have enough time, then phase two of the model estimates the probability of collision based on the surface area of the vehicle given the location of the animal within the trajectory. We demonstrate the utility of the model by estimating the probability of collision between a Canada goose and an approaching Boeing‐737 aircraft with the absence and presence of onboard lights of different wavelengths, a technological intervention aimed at minimizing bird strikes. Our model predicts that when a Canada goose is within the trajectory of a Boeing‐737, the average probability of collision is approximately 0.43; however, onboard lights with wavelengths tuned to the visual system of the species can reduce that probability on average by either 19% (red‐light onboard) or 32% (blue‐light onboard). The highest probability of collision occurred when the animal was in the center of the trajectory of the vehicle. The behaviors with the largest effect on reducing the probability of collision were an increase in flight‐initiation distance and an increase in escape speed. Our approach provides a framework to quantitatively predict how the probability of collision might change across different species, vehicles, and situations, which could be used in forecasting the impacts of present and future transportation projects on wildlife populations.

## INTRODUCTION

Globally, collisions between birds and aircraft pose a major safety hazard and monetary expense for the aviation industry (Allan, 2000; Altringer et al., 2021; Dolbeer et al., 2023). As air traffic is slated to increase with the proliferation of unoccupied aerial systems (i.e., UAS) (Davies et al., 2021; Federal Aviation Administration Aerospace Forecasts Fiscal Years 2024–2044, 2024; Mulero‐Pázmány et al., 2017), the frequency of bird and aircraft collisions, hereafter bird strikes, is expected to increase. At a time when most bird populations are globally declining (Burns et al., 2021; Lees et al., 2022; Rosenberg et al., 2019) and collisions with larger bodied species are simultaneously increasing (Dolbeer, 2020; Dolbeer et al., 2014), mitigating bird strikes has the potential to reduce both avian and human mortality as well as economic damage.

We know from the empirical literature that the behavioral response of an animal in close proximity of an approaching vehicle is critical in determining whether a collision does (i.e., the two come into contact) or does not occur (i.e., near miss), hereafter referred to as the probability of collision (Blackwell et al., 2019; Brieger et al., 2022; DeVault et al., 2015). Mathematical models exist to predict whether a prey animal can escape an approaching predator based on properties of their escape response (Bartashevich et al., 2024; Broom & Ruxton, 2005; Corcoran & Conner, 2016; Dill, 1974; Domenici, 2002; Kawabata et al., 2023; Ruxton et al., 2019). However, these models have yet to be applied to quantify how changes in the behavioral response of an animal affect the probability of collision with an approaching vehicle (DeVault et al., 2015; Guenin et al., 2024). Herein, we build upon existing models of predator–prey interactions to propose a novel model to estimate the probability of collision when an animal is in the path of an approaching vehicle.

Our study has three aims. First, we introduce a model that can be used to quantify the probability of collision considering variables such as escape trajectory, escape speed, vehicle size and vehicle speed. The model also incorporates new elements such as the location of the animal within the trajectory of the vehicle, and a stochastic component based on the sizes of the vehicle relative to the size of the animal. Second, we demonstrate the application of the probability of collision model to a scenario involving an approaching Boeing‐737 aircraft and a Canada goose (*Branta canadensis*), a large‐bodied, abundant, flocking species that can cause substantial damage upon collision (DeVault et al., 2018; Dolbeer et al., 2014). We parameterized our model with empirical data found in the peer‐reviewed literature of Canada goose escape responses to an approaching vehicle. Third, we then applied the model to a scenario where a Canada goose is approached by an aircraft but with onboard lights to investigate how different properties might affect the behavioral responses of Canada geese (Blackwell et al., 2012) and consequently the probability of collision.

Aircraft lighting of high chromatic contrast relative to the visual system of a target species has been proposed as a method to mitigate bird and aircraft collisions, especially beyond the airport boundary where mitigation methods are difficult to implement (Blackwell & Fernández‐Juricic, 2013; Dolbeer, 2011). Specifically, experimental evidence has shown that these onboard lights tuned to the avian visual system increase the distance a bird first detects an approaching aircraft, ultimately allowing more time for the animal to execute an escape response (Blackwell et al., 2009, 2012; Doppler et al., 2015). Additional evidence suggests that lights might also facilitate a more effective escape response by promoting avoidance (Goller et al., 2018; Lunn et al., 2023). However, to date, no quantitative estimates have been made for how changes in behavior caused by lights might reduce the probability of collision between a bird and an aircraft.

## METHODS

### Probability of collision model overview

Our mathematical model for the probability of collision is based on a series of equations separated into two distinct phases. Phase one calculates whether the animal has enough time to escape the trajectory of the approaching vehicle (Equations (1), (2), (3), (4), (5), Table 1). If the animal has enough time to escape the trajectory of the vehicle, then a collision is avoided. However, if the animal does not have enough time to clear the trajectory of the vehicle, then a collision is possible. Phase one assumes that the animal is within or near the trajectory of the vehicle and therefore a collision is possible, and that the trajectory of the vehicle is fixed (Box 1: Assumptions 1 and 2). Phase two assigns the animal some probability of collision depending on the location of the animal within the trajectory of the vehicle (Equations (6a), (6b), (6c)). We applied the model to estimate the probability of collision using a combination of specific and simulated parameter values that consider a variety of different scenarios where a high‐speed aircraft (i.e., a Boeing‐737) approaches a Canada goose. We built our model and made all estimates in R programing (version 4.3.2).

**TABLE 1:** A list of all variables, their corresponding symbols, definitions, and units used for Equations (1)–(6).

Symbol	Definition	Units
$T_{a}$	Time needed for the animal to escape the trajectory of the vehicle	s
$T_{v}$	Time until the vehicle reaches the location of the animal after escape initiation	s
$D_{\min}$	Distance to the edge of the trajectory of the approaching vehicle	m
l	Body length of the animal	m
$D_{\text{safe}}$	The total distance the animal needs to travel to reach safety (Equation 1)	m
$S_{a}$	Escape speed of the animal	m/s
θ	Escape angle ranging from 0° to 180°, where 0 is directly toward and 180° is directly away from the approaching vehicle	deg
δ	The time needed for the animal to reorient and initiate escape	s
$D_{\mathrm{FID}}$	Flight‐initiation distance or distance between the animal and the aircraft when the animal initiates escape	m
$S_{v}$	Approach speed of vehicle	m/s
$D_{\text{height}}$	The maximum height of the approaching vehicle	m
$D_{\text{width}}$	The maximum width of the approaching vehicle	m
$D_{\text{initial}}$	The animal's initial position within the vehicle's trajectory relative to vehicle width (Equation 4)	m
$D_{\text{collision}}$	The position of the animal within the trajectory of the vehicle when the vehicle reaches the location of the animal (Equation 5)	m
$A_{\text{front}}$	The frontal surface area of the approaching aircraft	m^2^

BOX 1 **A list of the model assumptions**

1. The vehicle is approaching directly, alternatively the animal is within the path trajectory of the vehicle.
2a. The trajectory of the vehicle is linear and constant.
2b. Vehicle approach speed ($S_{v}$) is constant.
3a. The trajectory of the animal is linear and constant after it initiates escape.
3b. Animal escape speed is constant ($S_{a}$) after it initiates escape.
4. The animal can be located at any altitude within the trajectory of the vehicle.
5. Per the light application exercise, toward escape angles are paired with shorter flight‐initiation distances as part of a larger attraction response to the light stimuli.

### Phase one

**FIGURE 1:**
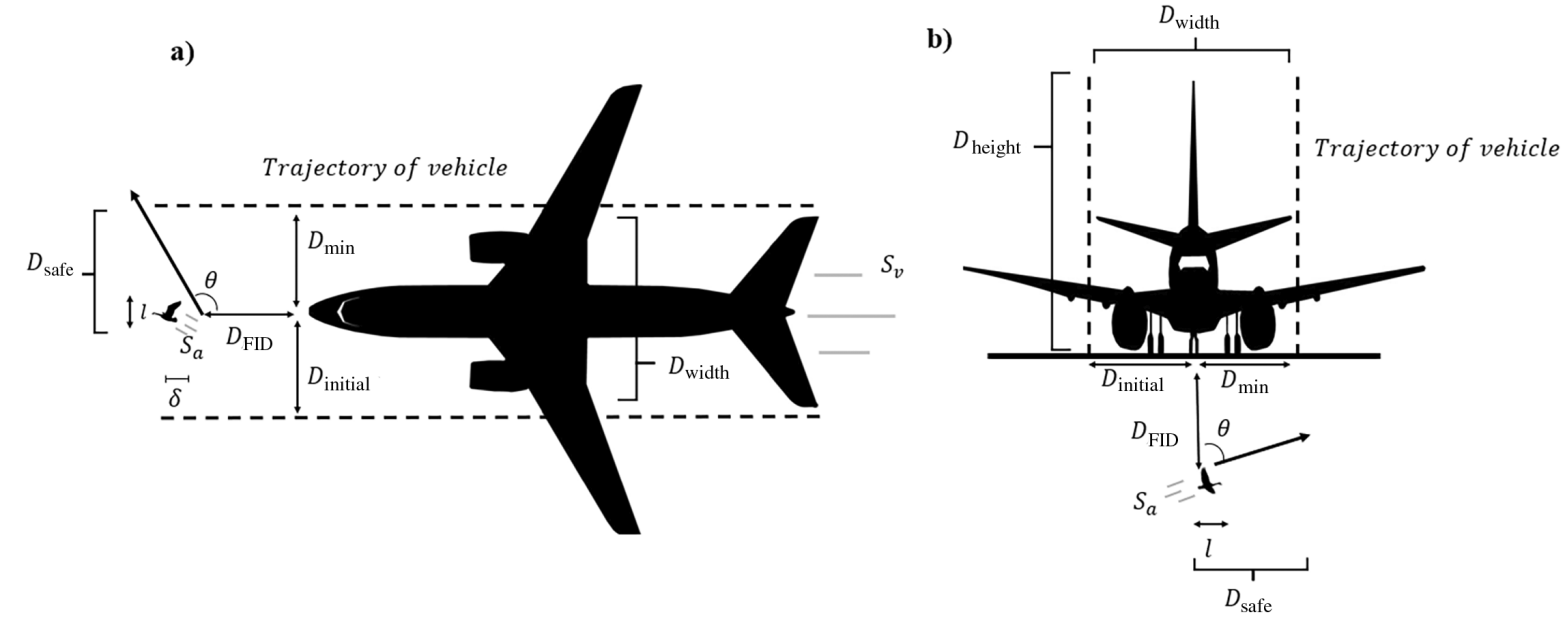
A top‐down (a) and front‐view (b) illustration of the variables considered in both Phase 1 and 2 of the model. The definitions for each parameter are provided in Table 1. The illustrations were created by Ryan B. Lunn based on the schematics provided in document D6‐58325‐6, https://www.boeing.com/commercial/airports/plan‐manuals.

Phase one rearranges the classic formula for speed (i.e., $\text{Speed}=\frac{\text{Distance}}{\text{Time}}$) and incorporates additional parameters to determine whether a collision is possible. Functionally, in phase one, we estimate the time that the animal needs to escape the trajectory of the vehicle ($T_{a}$) and the remaining amount of time until the vehicle reaches the location of the animal after escape is initiated ($T_{v}$) (Table 1). If the time the animal needs to escape ($T_{a}$) is greater than or equal to the amount of time remaining prior to the vehicle reaching the animal ($T_{v}$), then the situation results in a potential collision $(T_{a}$ ≥ $T_{v})$. Alternatively, if the time the animal needs to escape ($T_{a}$) is less than the time remaining prior to the arrival of the vehicle ($T_{v}$), then a collision is avoided $\left(T_{a}<T_{v}\right)$. The time needed to escape the trajectory of the vehicle ($T_{a}$) depends on the distance the animal needs to travel to safety ($D_{\min}$), the body length of the animal (l), escape speed ($S_{a}$), escape angle (θ), and sensory‐motor delays as the animal reorients and begins to accelerate (δ) (Table 1, Figure 1). After initiating escape, the animal needs time to travel some minimum distance to safety ($D_{\min}$) and additionally travel beyond its own body length (l) to completely avoid a collision, where the maximum possible value of $D_{\min}$ is the entire width of the vehicle (Equation 1).

(1) $$D_{\text{safe}}=D_{\min}+l.$$

$D_{\text{safe}}$ is the total distance the animal needs to travel to clear the trajectory of the vehicle.

Animals often combine a mixture of protean and optimal escape trajectories, alternatively escape angle (θ), to successfully escape or avoid an approaching threat, such as a natural predator (Domenici, 2002; Kawabata et al., 2023; Kimura & Kawabata, 2018; Walker et al., 2005). Escape angles (θ) are typically defined relative to the approach angle of the threat. Herein we define 0° as flight directly toward the approaching vehicle and 180° as flight directly away from the vehicle, where escape angles are limited between a range of 0° and 180°. Escape angles that differ from a perpendicular escape angle (i.e., 90°) extend the time needed for the animal to completely cross $D_{\text{safe}}$. Additionally, $T_{a}$ is dependent upon the escape speed of the animal ($S_{a}$) and the additional time required to reorient and accelerate as it enacts its escape response (δ) (Equation 2; Figure 1).

(2) $$T_{a}=\frac{\frac{D_{safe}}{\sin\left(\theta \right)}}{S_{a}}+\delta$$

Equation (2) assumes a constant escape speed by the animal (Box 1: Assumption 3).

The time remaining until the vehicle reaches the location of the animal after escape initiation ($T_{v}$) depends on the flight‐initiation distance ($D_{\mathrm{FID}}$), escape speed ($S_{a}$), and escape angle (θ) of the animal, and the approach speed of the vehicle ($S_{v}$) (Table 1, Figure 1). After the animal initiates its escape response ($D_{\mathrm{FID}}$), it has a limited amount before the vehicle reaches the location of the animal depending on the approach speed of the vehicle ($S_{v}$). However, depending on the escape speed ($S_{a}$) and angle (θ) of the animal, the vehicle will reach the position of the animal either relatively sooner or later as the animal moves either farther away or closer to the approaching vehicle (Equation 3).

(3) $$T_{v}=\frac{D_{\mathrm{FID}}}{S_{v}+\left(\cos\left(\theta \right)\times S_{a}\right)}$$

Equation (3) assumes a constant vehicle approach speed $\left(S_{v}\right)$ and animal escape speed $\left(S_{a}\right)$ (Box 1: Assumptions 2 and 3).

Consequently, if $T_{a}$ (i.e., time needed to escape) is greater than or equal to $T_{v}$ (i.e., time remaining to successfully escape) a collision is possible. If a collision is possible then phase two of the model estimates some probability of collision based on the location of the animal within the trajectory of the vehicle. However, phase two is not applicable to situations where $T_{a}$ < $T_{v}$ because a collision is entirely avoided, assuming the animal does not change directions or stop (Box 1: Assumption 3).

### Phase two

Phase two has two distinct components: (1) an estimate of the location of the animal within the trajectory of the vehicle at the moment of collision ($D_{\text{collision}}$) and (2) the assignment of the probability of collision based on that location (i.e., $P\left(\text{Collision}\right)$).

We estimated $D_{\text{collision}}$ based on the absolute minimum distance to safety ($D_{\min}$), the entirety of the vehicles width ($D_{\text{width}}$), the escape speed ($S_{a}$) and angle (θ) of the animal, and the duration of time that elapsed since the animal initiated escape ($T_{v}$). First, the minimum distance to safety ($D_{\min}$) and the trajectory width of the vehicle $\left(D_{\text{width}}\right)$ are used to estimate the initial position of the animal in the trajectory of the vehicle at the time when escape is initiated ($D_{\text{initial}}$) (Table 1, Figure 1, Equation 4).

(4) $$D_{\text{initial}}=D_{\text{width}}-D_{\min}$$

From the initial location of the animal $D_{\text{initial}}$, the model estimates how much further the animal travels within the trajectory of the vehicle while the vehicle continues to approach by multiplying escape speed $\left(S_{a}\right)$, angle $\left(\theta \right)$, and the time remaining since escape initiation ($T_{v}$, see Equation 3), which yields Equation (5).

(5) $$D_{\text{collision}}=D_{\text{initial}}+\left(\cos\left(\theta \right)\times S_{a}\times T_{v}\right)$$

$D_{\text{collision}}$ is the location, specifically the midpoint, of the animal in the trajectory of the vehicle the moment the vehicle reaches the animal.

**FIGURE 2:**
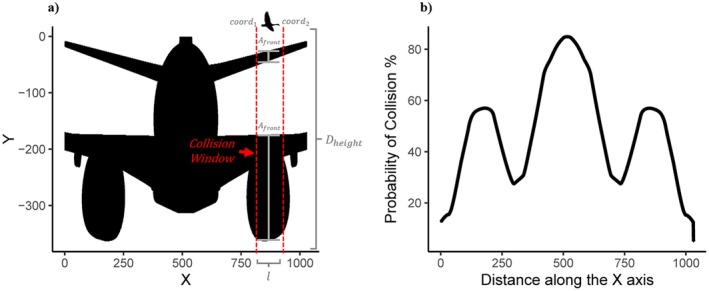
(a) The schematic of a Boeing‐737 aircraft used to estimate the frontal surface area. The red, dotted line represents the *x*‐axis intercepts of both ${\text{coord}}_{1}$ and ${\text{coord}}_{2}$ that define the location of the collision window on the *x*‐axis. (b) The sum of all pixels per the entirety of the goose's body length and for each potential location of the goose along the *x*‐axis of the trajectory of the aircraft. The right side shows the decrease in the probability of collision as the gooses body length exits the final edge of the trajectory. The illustrations were created by Ryan B. Lunn based on the schematics provided in document D6‐58325‐6, https://www.boeing.com/commercial/airports/plan‐manuals.

Estimates of $P\left(\text{Collision}\right)$ are based on the frontal surface area of the vehicle $\left(A_{\text{front}}\right)$ at the point of contact, location of the animal in the trajectory of the vehicle ($D_{\text{collision}}$), and body length of the animal $\left(l\right)$ (Table 1). We defined the trajectory of the vehicle as a 2D planar space bound by vehicle width $\left(D_{\text{width}}\right)$ and height $\left(D_{\text{height}}\right)$, which, respectively, can be thought of as the *x* and *y* axes (Figure 2). We estimated the probability of collision as the ratio between all the space occupied by the frontal surface area of the vehicle $\left(A_{\text{front}}\right)$ and the entire surface area at that location on the *x*‐axis where the animal could be (i.e., product of l and $D_{\text{height}}$), assuming the animal (i.e., a bird) could be at any random altitude (i.e., *y*‐axis) within the collision window (Box 1: Assumption 4; Equations (6a), (6b), (6c)).

(6a) $${\text{coord}}_{1}=D_{\text{collision}}-\left(\;\frac{l}{2}\;\right)$$

(6b) $${\text{coord}}_{2}=D_{\text{collision}}+\left(\;\frac{l}{2}\;\right)$$

(6c) $$P\left(\text{Collision}\right)=\frac{1}{D_{\text{height}}\times l}\int_{{\text{coord}}_{1}}^{{\text{coord}}_{2}}A_{\text{front}}$$

### Parameter selection and simulation approach

We applied our model to estimate the probability of collision for a scenario where a high‐speed aircraft (i.e., a Boeing‐737) approaches a Canada goose. We investigated how the probability of collision changes by systematically iterating through a range of realistic values for four different parameters: goose escape speed ($S_{a}$), sensory‐motor delay $\left(\delta \right)$, the minimum distance to safety $\left(D_{\min}\right)$, and aircraft approach speed ($S_{v}$) (Table 1). Herein, we used only a single value for body length (l), where we defined body length as the linear distance from the tip of the beak to the outer edges of the tail feathers (115 cm, Bellrose, 1976).

We varied escape speed ($S_{a}$) between 1 and 17 m/s, in increments of 2 m/s, based on the recorded flight speed of Canada geese (Wege & Raveling, 1984). The sensory‐motor delay values $\left(\delta \right)$ varied between 0 and 1 s in increments of 0.1 s based on the observed response delays of different bird species (Guenin et al., 2024; Provini et al., 2012). A sensory‐motor delay of 0 s represents a scenario where the animal is actively moving when it crosses into the trajectory of the vehicle, whereas 1 s means the animal took an entire second before it began to move. The minimum distance to safety ($D_{\min}$) varied from 1 to 14.35 m by increments of 1.48 m for a total of 10 different intervals. We chose 14.35 m as the maximum distance to safety based on the width between the edges of the horizontal stabilizers of a 737 commercial aircraft (Figure 2). We elected not to use the entire width of the aircraft wingspan because the collisions with the highest probability of damage, once a collision occurs, are impacts to the fuselage or engine ingestion (Dolbeer et al., 2023; Liu et al., 2019) (Figure 2). Lastly, we varied aircraft approach speed ($S_{v}$) between 70.47 (i.e., 150 kts) and 270.97 m/s by increments of 14.32 m/s for a total of 15 different approach speeds based on the range of Federal Aviation Administration recommended approach speeds that occur at different flight phases (e.g., take‐off run, climb, cruise, approach, landing) (Instrument Procedures Handbook: FAA‐H‐8083‐16A, 2017).

For each run of the model, we simulated a single flight‐initiation distance ($D_{\mathrm{FID}}$) and escape angle (θ). We simulated flight‐initiation distance values $\left(D_{\mathrm{FID}}\right)$ from a normal distribution with a mean of 56.2 m and a SD of 16.5 m, based on Canada goose escape responses to direct approaches by an automobile (i.e., a truck) (Blackwell et al., 2019). Escape angle values (θ) were simulated from one of two different uniform distributions. Birds either received an escape angle (θ) from a uniform distribution with escape angles (θ) ranging from 0.01° to 89.99°, hereafter referred to as the “toward” distribution, or from a uniform distribution with escape angles (θ) ranging from 90.01° to 179.99°, hereafter the “away” distribution. In our parametrization of the model, geese had a different probability of receiving either an escape angle from the “toward” (0.58) or “away” (0.42) distribution based on the frequency of different Canada goose behavioral responses reported by pilots obtained from the “Remarks” section of the Federal Aviation Administration wildlife collision database (Appendix S1).

To account for variation in model predictions attributable to differences in simulated flight‐initiation distance ($D_{\mathrm{FID}}$) and escape angle (θ) values, we ran the model with 500 iterations for each unique combination of parameter values.

### Estimating the probability of collision

We quantified the probability of collision by digitizing a to‐scale‐schematic of a Boeing‐737 (Figure 2). We estimated the frontal surface area of the vehicle ($A_{\text{front}}$) by summing all the pixels occupied by the aircraft (Figure 2), divided by the total number of pixels within a specific subsection along the *x*‐axis defined by the length of the goose and height of the aircraft, hereafter referred to as the “collision window.” We converted the length of the goose to pixels by first dividing the width of the trajectory (i.e., 14.35 m) by the width of the aircraft schematic image (i.e., 1031 pixels) (Figure 2) to estimate the m/pixels conversion factor (0.0139 m/pixels) and then divided goose length (l) (i.e., 1.15 m) by the conversion factor to estimate the length of a goose in nearest whole pixels (83 pixels).

### Model application

We applied our model to quantify how lights of different wavelengths onboard an approaching aircraft might affect the probability of collision through altering the escape behavior of Canada geese. Lights have been shown to lead to earlier alert responses to approaching vehicles (Blackwell et al., 2012). Specifically, Canada geese alerted 4.1 s earlier to an approaching aircraft with a light turned on (11.4 ± 4.4 s) relative to an aircraft with a light turned off (7.3 ± 4.4) (Blackwell et al., 2012). This early alert provides an opportunity for the animal to escape sooner, thus expanding the range of potential flight‐initiation distances ($D_{\mathrm{FID}}$) (Blackwell & Fernández‐Juricic, 2013). Consequently, for an aircraft approaching with lights on, we simulated flight‐initiation distance ($D_{\mathrm{FID}}$) values from a uniform distribution with a minimum of 0 m and maximum flight‐initiation distance ($D_{\mathrm{FID}}$) based on the maximum possible alert distance. We estimated the maximum possible alert distance by multiplying the temporal benefit provided by the light source, hereafter β, by the aircrafts approach speed. We systematically varied the temporal benefit of the light source (β) from 0.5 to 8.5 s by increments of 0.89 s for 10 different intervals, based on the mean observed temporal benefit (4.1 s) and SD in alert time (4.4 s) observed in Blackwell et al. (2012).

Light wavelengths of high chromatic contrast (i.e., a 483 nm “blue light” and 631 nm “red light”) have also been shown to affect the avoidance responses in Canada geese in a single choice experiment (i.e., a T‐maze test with a lighton on one side and a light turned off on the other side), where differences in the probability of avoidance could potentially translate to differences in the escape angle of the animal (Lunn et al., 2023). Lunn et al. (2023) found that after repeated exposures, geese tended to avoid a 483‐nm light (i.e., blue) (probability of avoidance 0.65) and were attracted to 631 nm light (i.e., red) (probability of avoidance 0.11). Consequently, we explored how the probability of collision changes for different wavelengths of high chromatic contrast (i.e., a blue‐ and red‐light) assuming an avoidance response translates to an escape trajectory away from the approaching aircraft (i.e., an escape angle $\left(\theta \right)$ > 90°). Specifically, in a blue‐light scenario geese had a 65% chance of receiving an escape angle from the “away” distribution, where in a red‐light scenario geese had a 11% chance of receiving an escape angle from the “away” distribution according to the difference in the probability of avoidance reported in the final trials of Lunn et al. (2023). Presently, there are no data on how white light affects the probability of avoidance for Canada geese and consequently our model application exclusively focuses on red and blue wavelengths of light.

No study that we are aware of has examined how different wavelengths of light simultaneously affect different combinations of flight‐initiation distance ($D_{\mathrm{FID}}$) and escape angles (θ). The findings of Lunn et al. (2023) indicate that geese developed an attraction response to the red light but showed a mild avoidance response to the blue‐light after repeated exposures. We assumed for both the blue‐ and red‐light scenarios that “toward” escape angles are indicative of a potential attraction response to the lights (not necessarily to the approaching aircraft) and thus paired them with shorter flight‐initiation distances ($D_{\mathrm{FID}}$), whereas “away” escape angles were indicative of avoidance and thus paired with longer flight‐initiation distances $\left(D_{\mathrm{FID}}\right)$. Specifically, the presence of attractants such as a food source, potential mate, or flock members results in shorter flight‐initiation distances, where the presences of an aversive stimuli or repellent associated with greater perceived risk, such as faster and more direct predator approaches, results in longer flight‐initiation distances (Blackwell et al., 2019; Cooper, 2009a; Hammer et al., 2025; Ventura & Galdino, 2021; Ydenberg & Dill, 1986). Additionally, birds can either be attracted or repelled by different light stimuli (Adams et al., 2021; Poot et al., 2008; Rodríguez et al., 2017; Van Doren et al., 2021), potentially leading either to shorter or longer flight‐initiation distances, respectively.

For both the blue‐ and red‐light scenarios if the simulated escape angle (θ) was received from the “toward” distribution then consequently the flight‐initiation distance $\left(D_{\mathrm{FID}}\right)$ was simulated from the same distribution as a no‐light scenario (i.e., normal distribution, mean = 56.2 m, SD = 16.5) and not affected by β. However, if an escape angle $\left(\theta \right)$ was received from the “away” distribution, then consequently flight‐initiation distance values were simulated from the distribution affected by β (i.e., a uniform distribution ranging from 0, to $S_{a}\times$ β).

### Reporting results

We generated a total of 222,750,000 predictions of whether a collision would or would not occur with our model for all combinations of goose escape speed ($S_{a}$), sensory‐motor delays $\left(\delta \right)$, the minimum distance to safety $\left(D_{\min}\right)$, aircraft approach speed ($S_{v}$), and the three different light scenarios (i.e., no light, blue light, red light) which included the temporal benefit (β) afforded by a light onboard. For each combination of parameters, we generated 500 predictions with different simulated values for flight‐initiation distance ($D_{\mathrm{FID}}$) and escape angle (θ).

**FIGURE 3:**
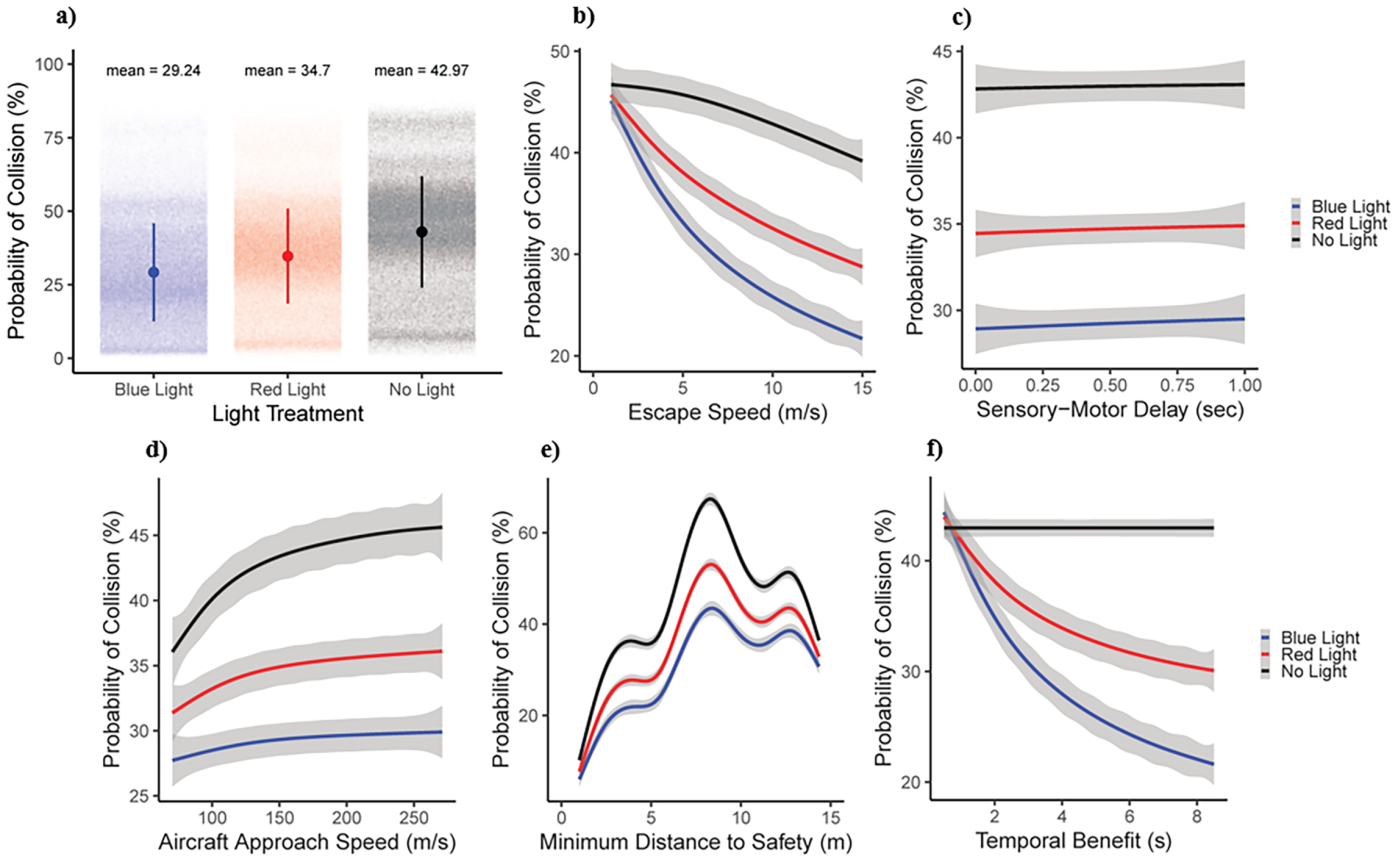
The relationship between each variable manipulated in our model and the probability of collision, where gray bars represent the SD. (a) The mean and SD in the probability of collision for each of the three light scenarios, where each point represents the average probability of collision for over 500 simulation runs with the same combination of manipulated variables. (b) The relation between escape speed (in meters per second) and probability of collision for the three light scenarios. (c) The relation between sensory‐motor delay (in seconds) and probability of collision for the three light scenarios. (d) The relation between aircraft approach speed (in meters per second) and probability of collision for the three light scenarios. (e) The relation between the minimum distance to safety (in meters) and probability of collision for the three light scenarios. (f) The relation between the temporal benefit afforded by the light (in seconds) and probability of collision for the three light scenarios.

**FIGURE 4:**
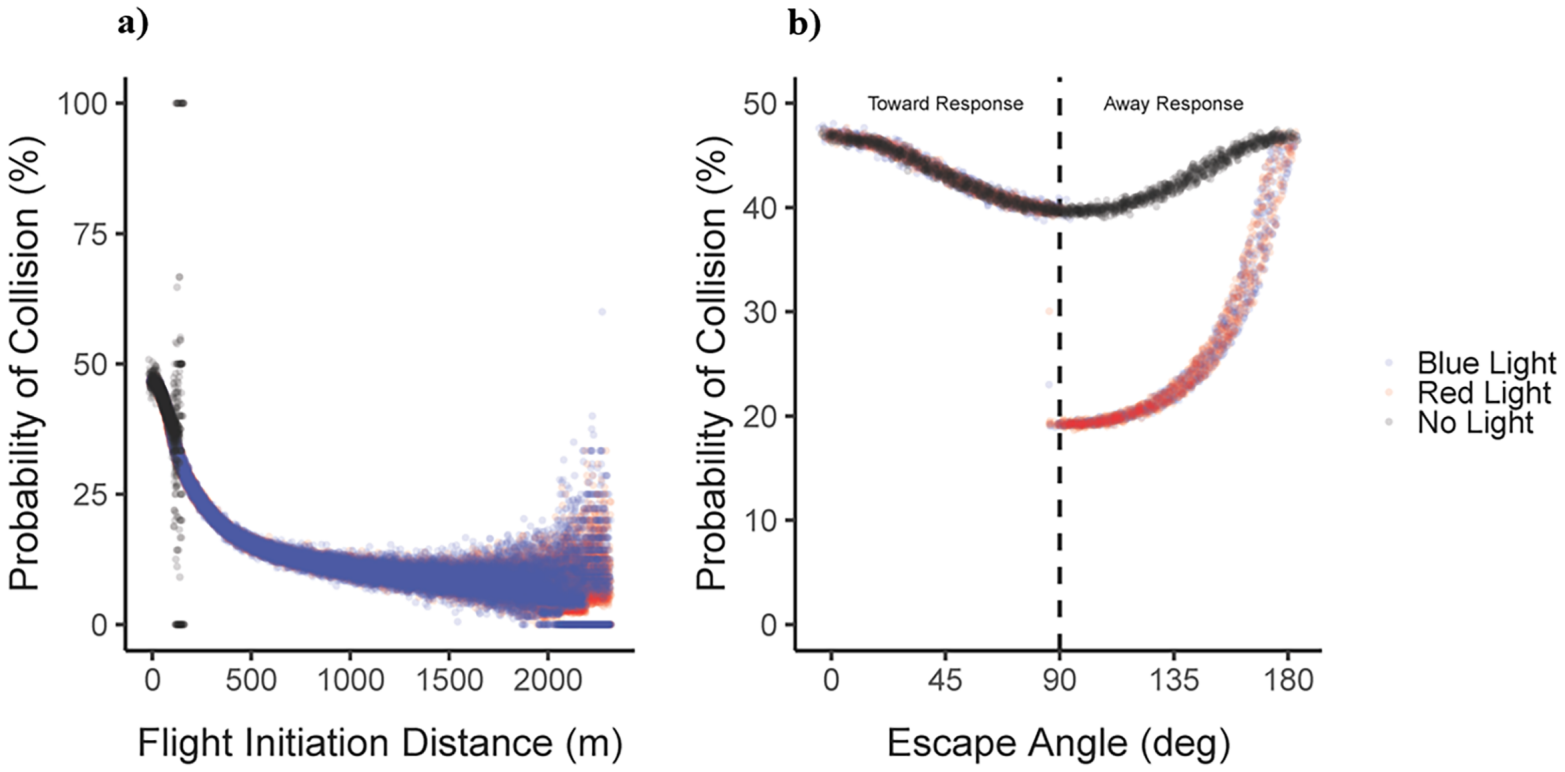
(a) Plot of the average probability of collision for all model predictions made with a specific flight‐initiation distance value (in meters) separated by the three light scenarios. (b) Plot of the average probability of collision for all model predictions made with a specific escape angle value (in degrees), separated by the three light scenarios.

We present the model predictions for the probability of collision in Figures 3 and 4, where both figures show the relationship between the probability of collision and a single variable. In Figure 3, the *x*‐axes were variables that we systematically manipulated, whereas in Figure 4, the *x*‐axes are simulated variables (see above). We estimated the predicted probability of collision in Figure 3 by first summing the number of collisions that occurred for each unique combination of parameters and then divided the total by 500, representing the number of different runs of the model with simulated flight‐initiation distance ($D_{\mathrm{FID}}$) and escape angle (θ) values. We estimated the predicted probability of collision in Figure 4 by first binning both flight‐initiation distance ($D_{\mathrm{FID}}$) and escape angle (θ) to within 0.1 of either a meter or degree and then added the total number of collisions that occurred within that bin divided by the total number of predictions within that bin. To describe the relationship between our continuous variables and the probability of collision regarding the three different light scenarios, we fitted a curve from a general additive model using the *geom_smooth* in the *ggplot2* package (Wickham et al., 2016). The results of each figure can be interpreted as the average effect of that single parameter value on the probability of collision across all other possible parameter values.

## RESULTS

### Overview

First, the mean probability of collision among all combinations of parameters was the lowest for the blue light scenario (mean ± SD, 0.292 ± 0.167), with an increase for the red‐light scenario (0.347 ± 0.161) and with the highest probability of collision occurring for the no‐light scenario (0.429 ± 0.189) (Figure 3a). Second, an increase in escape speed of the animal ($S_{a}$) generally resulted in a nonlinear asymptotic decrease in the probability of collision (Figure 3b). For the blue‐ and red‐light scenarios, the probability of collision decreased at a decreasing rate with escape speed. In contrast, the no‐light scenario yielded a probability of collision that decreased but at an increasing rate with increasing escape speed (Figure 3b). As sensory‐motor delays increased, the probability of collision increased linearly and slightly, with a similar pattern across all three light scenarios (Figure 3c). An increase in aircraft approach speed resulted in the probability of collision increasing to an asymptote for each light scenario (no light > red light > blue light; Figure 3d). The relationship between the minimum distance to safety and the probability of collision was multimodal with three different inflection points (Figure 3e). Generally, as the minimum distance to safety increased, so did the probability of collision. However, the shape of the curve strongly reflected the difference in the probability of collision based on the shape of the aircraft and the location of the animal within the trajectory. Specifically, the probability of collision was highest when the location of the animal within the trajectory of the aircraft coincided with either the fuselage or the engines, where this pattern was similar for all three light scenarios (Figure 3e). Also, as the temporal benefit afforded by a light source increased, the probability of collision decreased to an asymptote and was only relevant for the blue‐ and red‐light scenarios (Figure 3f).

Larger flight‐initiation distances ($D_{\mathrm{FID}}$) resulted in dramatically lower probabilities of collision for both the blue‐ and red‐light scenarios (Figure 4a). Additionally, escape angles (θ) closer to 90° (i.e., escaping perpendicularly to the approaching vehicle) had a lower probability of collision relative to escape angles $\left(\theta \right)$ closer to either 0° or 180° (Figure 4b). The “away” escape angles for both the blue‐ and red‐light scenarios (i.e., paired with longer flight‐initiation distance ($D_{\mathrm{FID}}$)) yielded a lower probability of collision, in contrast to “toward” escape angles (Figure 4b).

## DISCUSSION

To summarize, we built upon an existing theoretical foundation (Domenici, 2002; Kawabata et al., 2023) to propose a mathematical model to estimate the probability of collision an animal faces given it is within the trajectory of an approaching aircraft. Our model quantifies how differences in specific aspects of an escape response in the final seconds prior to the arrival of a high‐speed approaching vehicle affects the probability of collision. We then applied the model to quantify how a given technological intervention (i.e., onboard light stimuli) aimed at altering the escape response of the animal might subsequently affect the probability of collision. Our model demonstrates the importance of considering how animals simultaneously alter multiple properties of an escape response to reduce the probability of collision, how the probability of collision varies depending on the location of the animal within the trajectory of the vehicle, and the potential onboard lighting tuned to eyes of the target species has to reduce the probability of collision.

Animals often rely on a combination of several different sequential behaviors to reduce the mortality risk associated with an approaching predator (Branco & Redgrave, 2020; Evans et al., 2019). Evidence suggests that animals rely on similar behaviors when attempting to avoid approaching vehicles (DeVault et al., 2015; Lima et al., 2015; Lunn et al., 2022). Specifically, individuals can adjust their flight‐initiation distance (Cooper & Blumstein, 2015; Stankowich & Blumstein, 2005; Ydenberg & Dill, 1986), escape speed (Domenici & Blake, 1991; Lind et al., 2002), escape angle (Domenici et al., 2011a, 2011b; Domenici & Blake, 1993), or opt not to escape at all (Blackwell et al., 2020; Broom & Ruxton, 2005; Cooper, 2009b) to reduce mortality risk from an approaching threat (Caro, 2005).

The model can be used to identify the components of an escape response that have the largest effect on the probability of collision. Technological interventions or management can then target these specific behaviors of relatively larger effect size to enact larger reductions in the probability of collision. For instance, in the case of Canada geese and an approaching 737 with no‐light onboard, flight‐initiation distance had the largest effect, where an increase from 0 m (0.460) to greater than 120 m (0.361) resulted in a 21.6% decrease in the probability of collision (Figure 4a). Differences in escape speed had the second largest effect, where an increase from 0 (0.467) to 17 m/s (0.377) resulted in a 19.3% decrease in the probability of collision (Figure 3b). Lastly, difference in escape angle from 0° (0.467) or 180° (0.464) to 90° (0.397) (Figure 4b), respectively, resulted in a 14.9% and 14.4% decrease in the probability of collision. Thus, we would expect that a strategy aimed at increasing Canada goose flight‐initiation distance or escape speed would yield the largest reductions in the probability of collision relative to an approaching 737.

**FIGURE 5:**
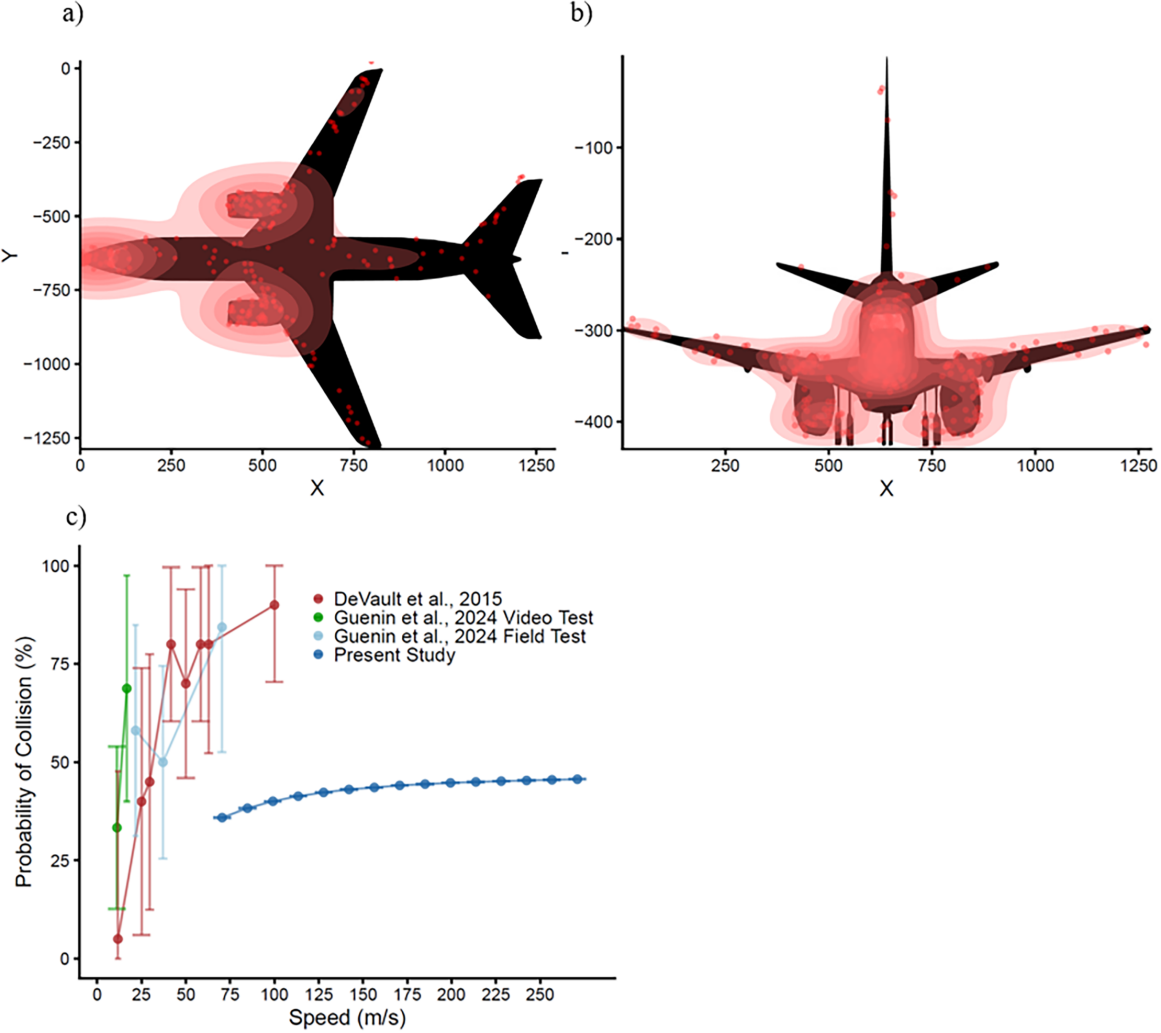
(a, b) A density map of the collision locations reported in the Federal Aviation Administration (FAA) wildlife strike database between Canada geese and a 737 aircraft, where (a) is the frontal view of an aircraft and (b) a top‐down view of the aircraft. (c) The relationship between approach speed and the model‐predicted probability of collision compared to the empirical relationship between observed probability of collision and vehicle approach speed reported in DeVault et al. (2015) and Guenin et al. (2024). The illustrations were created by Ryan B. Lunn based on the schematics provided in document D6‐58325‐6, https://www.boeing.com/commercial/airports/plan‐manuals.

Our model also enables us to quantify how different vehicle approach scenarios affect the probability of collision, which can aid in developing collision mitigation strategies. For example, in the case of a 737 approaching a Canada goose, the location of the animal in the trajectory of the vehicle had the largest effect, where the difference between a minimum distance to safety of 1 m (0.102) versus 8.42 m (0.673) resulted in a 570% increase in the probability of collision. Specifically, the three inflection points (i.e., peaks) in Figure 3e correspond to the aircraft engines and fuselage, where the probability of collision increases due to their larger frontal surface areas. The predictions of our model align with the observations of the Federal Aviation Administration's wildlife strike database, where the most frequently struck locations of Canada geese on a 737 are the engines and the fuselage (*n* = 193, Federal Aviation Administration Wildlife Strike Database, 2024) (Figure 5a,b). A potential mitigation strategy therefore might focus on deterring birds away from sections of the aircraft with the largest frontal surface area.

Our model also provides a quantitative framework to make predictions about high‐speed vehicle approach scenarios that are difficult to test empirically. Previously the fastest simulated approach speed empirically tested was 100 m/s by DeVault et al. (2015) (Figure 5c). In our example with a Canada goose and a Boeing‐737, we were able to estimate that the probability of collision increased by 27% from 70 (0.359) to 271 (0.457) m/s, extremely fast approach speeds. Additionally, previous empirical studies have been limited to estimating whether a collision occurs based on only a few properties of an escape response (i.e., flight‐initiation distance) and approaching vehicle (i.e., approach speed, vehicle width) (DeVault et al., 2015; Guenin et al., 2024). Our model proposes a framework for the additional variables that need to be collected to more accurately predict the outcome of an animal–vehicle interaction.

Compared to the empirical data the predictions of our model align qualitatively (i.e., an increase in approach is associated with an increase in the probability of collision; DeVault et al., 2015, Guenin et al., 2024). However, the disparity in the probability of collision estimates between empirical and theoretical approaches (Figure 5c) is most likely due to a combination of two factors. First, our study considers the probability of collision with an approaching aircraft where the empirical studies focus on the probability of collision relative to an approaching automobile. In our approach, the probability of collision varies from 0.102 to 0.673 depending on the location of the animal within the trajectory of the aircraft where the probability of collision was assumed to be 1 within the trajectory of an automobile. Second, our model considers how animals might alter many different components of their escape response simultaneously, such as escape angle and escape speed, not just flight‐initiation distance. In Figure 4a, as flight‐initiation distance continues to increase the probability of collision on average does decrease but is not inevitably 0%. Logically, even if an animal adopts an extremely long flight‐initiation distance, those responses still must be paired with an effective escape angle (the probability of collision varies from 0.397 to 0.467 or 0.464 depending on an escape angle of 0° or 180°) and escape speed (the probability of collision varies from 0.377 to 0.467) to avoid a collision. Differences in these components could be critical in determining whether an animal avoids a potential collision.

Both the probability of capture in the context of predator–prey interactions and the probability of collision in the context of animal–vehicle interactions rely on similar variables to quantify the outcome of an interaction with either a predator or a vehicle; however, additional assumptions and parameters are needed to estimate the probability of collision (Box 1). We argue that three major differences need to be considered. First, the probability of collision, especially for larger vehicles, is often not homogenous throughout trajectory of the vehicle (i.e., portions with larger frontal surface area increase the probability of collision). As a result, the specific location of the animal within that trajectory should be considered, because a collision still might be avoided despite the vehicle reaching the location of the animal (e.g., a goose passing over the fuselage of the aircraft, see Phase two & Figure 2 above).

Second, vehicles generally remain on a fixed and linear trajectory when approaching an animal as opposed to the dynamic trajectories of predators (Peterson et al., 2021). Models of predator–prey capture vary in whether they assume a predator approaches linearly or nonlinearly (Corcoran & Conner, 2016; Domenici, 2002; Kawabata et al., 2023). Vehicles are commonly limited to traveling on a designated substrate (i.e., road) or predetermined course (i.e., flight path), especially at faster speeds; therefore, when modeling animal–vehicle collisions generally a linear approach can be assumed. Yet, a fixed and linear trajectory does not necessarily mean that the behavior of the vehicle does not change (pilots alter flight paths, speeds, etc.). An important component for future research is to assess the effects of driver behavior seconds prior to a collision (e.g., Pakula et al., 2023).

Third, the probability of collision is dependent on the approach angle of the vehicle. In empirical studies, approach angles are often categorized as either direct or indirect, defined by whether the trajectory of the predator intersects or just simply bypasses the prey animal (e.g., Domenici, 2002; Stankowich & Blumstein, 2005). However, the threshold approach angle that distinguishes between direct versus indirect angles is generally not explicitly defined, primarily because predators can dynamically alter their approach angle based on prey escape trajectories as they get closer (Corcoran & Conner, 2016; Howland, 1974; Peterson et al., 2021). However, because vehicles are often limited to fixed trajectories (see above), we propose that the critical angle of a direct or indirect approach can be quantitatively defined as

(7) $$\theta_{threshold}=\sin^{-1}\left(\frac{D_{half+l}\;}{D_{\mathrm{mid}}}\right)$$

where $\theta_{\text{threshold}}$ is the critical approach angle differentiating between an indirect and direct approach at a given time point, $D_{\mathrm{mid}}$ is the distance between the center of the vehicle and the animal, and $D_{\text{half}}$ is half the maximum width of the vehicle, and l is the length of the animal. Specifically, vehicle approach angles greater than $\theta_{\text{threshold}}$ will equate to an indirect approach where if the animal does not enact an escape response a collision can be avoided, where approach angles less than or equal $\theta_{\text{threshold}}$ will result in a potential collision if no response is enacted. The probability of collision with a vehicle being completely dependent on approach angle might explain in part why some animals appear to adopt relatively shorter flight‐initiation distances (Blackwell et al., 2020; Holmes et al., 1993), not flee at all (Guenin et al., 2024), or flee after the vehicle has passed (Pfeiffer et al., 2025) because often the probability of collision is 0 for an indirect approach.

Importantly, Equation (7) does not explicitly affect the predictions of our model because Assumption 1 (Box 1) states the vehicle is approaching directly, and therefore the approach angle is below $\theta_{\text{threshold}}$ and a collision is possible, if no response is enacted. As computer vision technology becomes further integrated into automated vehicle navigation (i.e., advanced air mobility), estimating the vehicles approach angle ($\theta_{\text{threshold}}$) relative to a detected wildlife hazard could be important in monitoring the prevailing probability of collision and whether collision avoidance measures are necessary by the vehicle (Corcoran et al., 2021; Huijser et al., 2015; Nandutu et al., 2022).

The Federal Aviation Administration's wildlife strike database reports that interactions with Canada geese and aircraft over a 23‐year period have cost the airline industry approximately 183 million dollars, a 7.95‐million‐dollar annual cost (Dolbeer et al., 2023). Our model suggests that blue and red onboard lighting tuned to the eyes of Canada geese would reduce the probability of collision by about 14% and 8%, respectively, which potentially could have saved approximately 25 and 15 million US dollars over a 23‐year period for just one species. However, a more structured analysis is necessary to truly estimate the potential financial savings and variation in savings afforded by light stimuli following the approaches put forth by Altringer et al. (2021, 2024). Additionally, these estimates are based on assumptions about how animals change multiple aspects of their escape behavior simultaneously in response to a light stimulus, and future research needs to evaluate these assumptions.

Future efforts to quantify the probability of collision for an approaching vehicle can build upon this existing model in several concrete ways. First, our modeling approach can be applied to other vehicle types (rotorcraft, automobiles, boats, etc.) and other taxa of management or conservation concerns. Second, in our modeling approach, we did not incorporate any parameter or make any assumption about how animals delay escape after detection and continue to assess an approaching threat before initiating escape (Blumstein, 2010; Chan et al., 2010; DeVault et al., 2015; Guenin et al., 2024; Lunn et al., 2022). As such, the absolute value for the probability of collision estimates are most likely conservative (i.e., smaller), especially given the extremely fast range of speeds we used to model an approaching aircraft. Future studies quantifying the probability of collision should explicitly incorporate a risk assessment delay prior to flight‐initiation distance into estimates of the time needed for the animal to clear the path trajectory of the vehicle. Third, our model implicitly assumes that the altitude of the animal in the path trajectory of the vehicle is random because the animal could be at potentially at any height (Figure 2). In reality, the aircraft could be ascending or descending, and the bird as well could be attempting to gain or lose altitude to escape the trajectory of the vehicle. We did not assume any specific height within the trajectory of the vehicle because of the scarcity of empirical data to support a given range. However, if we had data on both take‐off velocity and take‐off or dive angle for Canada geese, we could improve the accuracy of the probability of collision estimates. Fourth, we acknowledge that the probability of collision varies in 3‐dimensional space depending on the fluid dynamics surrounding the aircraft, especially around the aircraft engines (Heimbs, 2011; Tatlıer & Baran, 2020). Computational fluid dynamics could be utilized in combination with the species flight speed to further improve estimates of the probability of collision and the probability of damage an aircraft might sustain upon impact.

We envision that our model be applied to quantitatively estimate the probability of collision for various species and different vehicle approach scenarios, which could ultimately aid in forecasting the impacts of present and future transportation projects on wildlife populations. Additionally, our model allows us to estimate the probability of specific components of the aircraft (fuselage, engines, wings, etc.) being struck, helping in the estimation of economic damage and safety hazards (Dolbeer et al., 2023). Overall, our framework can be used to develop targeted and preventative animal–vehicle collision mitigation strategies, especially as air‐traffic volume is forecasted to increase.

## AUTHOR CONTRIBUTIONS

Ryan B. Lunn contributed to conceptualization, methodology, data processing and analysis, visualization, writing‐original draft, writing‐review and editing, project administration. Bradley F. Blackwell contributed to funding acquisition, conceptualization, writing‐original draft, writing‐review and editing, and project administration. Esteban Fernández‐Juricic contributed to funding acquisition, conceptualization, methodology, data processing and analysis, writing‐original draft, writing‐review and editing, and project administration.

## CONFLICT OF INTEREST STATEMENT

The authors declare no conflicts of interest.

## Supporting information


Appendix S1.

## Data Availability

Data and code (Lunn & Fernandez‐Juricic, 2026) are available in the Open Science Framework at https://doi.org/10.17605/OSF.IO/ZH68X.
