# Supplementary material for: A model to quantify the probability of collision between birds and aircraft: Applications for onboard lighting
Source: Ecol Appl. 2026 Apr 1;36(3):e70227. doi: 10.1002/eap.70227 (PMC13041519; doi:10.1002/eap.70227)
Supplement: Supplementary file 1 — Appendix S1. [file EAP-36-e70227-s001.pdf]

Appendix S1

A model to quantify the probability of collision between birds and aircraft: Applications for onboard lighting

Ryan B. Lunn, Bradley F. Blackwell, Esteban Fernández-Juricic

Ecological Applications

Differences in whether a bird received an escape angle from the toward or away distribution were based on the behavior observed by pilots reported in the “Remarks” section of the Federal Aviation Administration’s Wildlife Strike database. We read through all pilot remarks from 2000 to 2025 (n=2204) for all reported wildlife hazard incidences involving a Canada goose recorded in the Federal Aviation Administration wildlife strike database. Of the 2204 reports only 138 described the gooses behavior or movement during the interaction. Of those 138 reports if the pilot described a scenario where the goose/geese moved either in front, towards, or across the aircrafts path we categorized those as “Toward” responses. If the pilot described a scenario where the goose/geese took flight, flushed, or veered away from the aircraft we categorized those as “Away” responses. The 138 remarks that were categorized as “Away” or “Towards” are available in the “Remarks_Exercise.csv” file in Lunn and Fernandez-Juricic (2026) at https://doi.org/10.17605/OSF.IO/ZH68X .

References

Lunn, R., and E. Fernandez-Juricic. 2026. A model to quantify the probability of collision between birds and aircraft: applications for onboard lighting. Open Science Framework. https://doi.org/10.17605/OSF.IO/ZH68X
